# The 1918–19 Influenza Pandemic in Boyacá, Colombia

**DOI:** 10.3201/eid1801.101969

**Published:** 2012-01

**Authors:** Gerardo Chowell, Cécile Viboud, Lone Simonsen, Mark A. Miller, Rodolfo Acuna-Soto, Juan M. Ospina Díaz, Abel Fernando Martínez-Martín

**Affiliations:** Arizona State University, Tempe, Arizona, USA (G. Chowell);; National Institutes of Health, Bethesda, Maryland, USA (G. Chowell, C. Viboud, M.A. Miller);; George Washington University, Washington, DC, USA (L. Simonsen);; Universidad Nacional Autónoma de México, Delegación Coyoacán, Mexico (R. Acuna-Soto);; Universidad Pedagógica y Tecnológica de Colombia, Boyacá, Colombia (J.M. Ospina Díaz, A.F. Martínez-Martín)

**Keywords:** influenza, 1918–19 pandemic, Boyacá, Colombia, transmissibility, excess deaths, mortality rates, age patterns, geography, deaths, viruses

## Abstract

To quantify age-specific excess-mortality rates and transmissibility patterns for the 1918–20 influenza pandemic in Boyacá, Colombia, we reviewed archival mortality records. We identified a severe pandemic wave during October 1918–January1919 associated with 40 excess deaths per 10,000 population. The age profile for excess deaths was W shaped; highest mortality rates were among infants (<5 y of age), followed by elderly persons (>60 y) and young adults (25–29 y). Mean reproduction number was estimated at 1.4–1.7, assuming 3- or 4-day generation intervals. Boyacá, unlike cities in Europe, the United States, or Mexico, experienced neither a herald pandemic wave of deaths early in 1918 nor a recrudescent wave in 1920. In agreement with reports from Mexico, our study found no death-sparing effect for elderly persons in Colombia. We found regional disparities in prior immunity and timing of introduction of the 1918 pandemic virus across populations.

## Abstract 2

Timing of pandemic onset and prior immunity of populations varied by region.

Quantitative analyses of age-specific death rates, transmissibility, and dissemination patterns of the 1918 influenza pandemic in the United States (*1**,**2*), Mexico (*3*), Peru (*4*), Japan (*5*), Europe (*6**,**7*), Taiwan (*8*), and Singapore (*9*) have shed light on the epidemiology of the most devastating pandemic in recent history (*10*). These studies revealed the pandemic’s unusual severity in young adults, occurrence in multiple waves, and higher transmission potential than that of seasonal epidemics (*11*). However, quantitative historical studies remain scarce for Latin America, Africa, and Asia, where our understanding of influenza disease patterns remains particularly weak.

The emergence of the pandemic influenza A (H1N1) 2009 virus in Mexico (*12**,**13*) reinforced the need to understand the epidemiology of past pandemics in the Americas to inform preparedness plans. We therefore analyzed death patterns for the 1918 influenza pandemic in Boyacá, a rural area in central Colombia, where influenza seasonality is less defined than in temperate regions (*14*). By using archival records, we quantified the age-specific excess-death rates and transmission potential of the 1918–19 pandemic in Boyacá and compared these findings with those reported for other locations, especially Mexico City, Mexico.

## Materials and Methods

### Study Location

**Figure 1:**
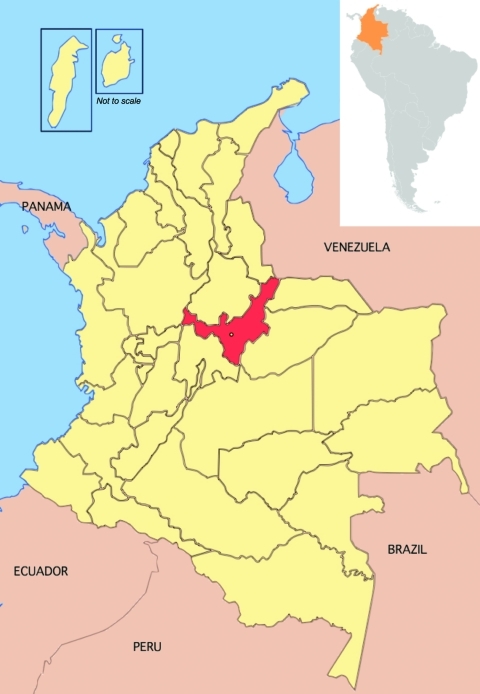
Colombia, showing Boyacá (in red) and other departments. Inset, location of Colombia within South America. Boyacá is located in the central part of Colombia within the Andes Mountain range and has a surface area of 8,630 km^2^. Insets show San Andrés Island (left) and Providencia Island. Figure adapted from http://en.wikipedia.org/wiki/Boyac%C3%A1_Department.

Boyacá is located in the central part of Colombia within the Andes Mountains at latitude ≈5.5°N (Figure 1). In 1918, the population of Boyacá was 659,947 and <50% of the area was occupied. Hygienic conditions were poor. A centralized disease notification system was lacking; however, death records were maintained by parishes.

The climate in Boyacá varies from high humidity and high mean temperature (≈40°C) in low areas near the Magdalena River (altitude 600 m) to cold mean temperature (<6°C) and permanent snow in the Cocuy Mountains (altitude 5,500 m). The 2 rainy seasons, April–May and October–November, produce ≈1,000 mm^3^/rainfall/year.

### Data Sources

#### Historical Death Records

**Figure 2:**
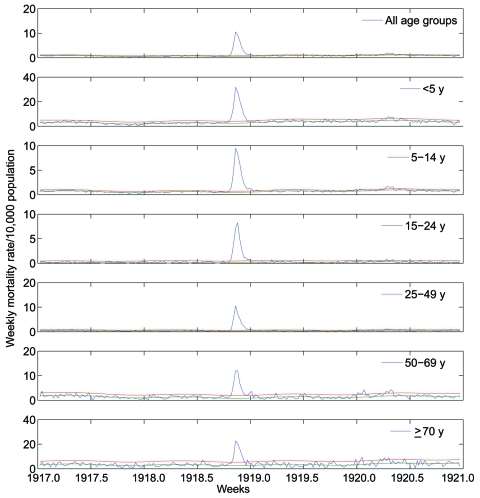
Age-stratified weekly respiratory mortality rates per 10,000 population in Boyacá, Colombia, 1917–1920. Background mortality rate derived from a seasonal regression model (blue); corresponding 95% CI curves are shown (red and green). Deaths in excess of the upper limit of the background mortality curve are deemed attributable to the 1918–19 influenza pandemic.

**Figure 3:**
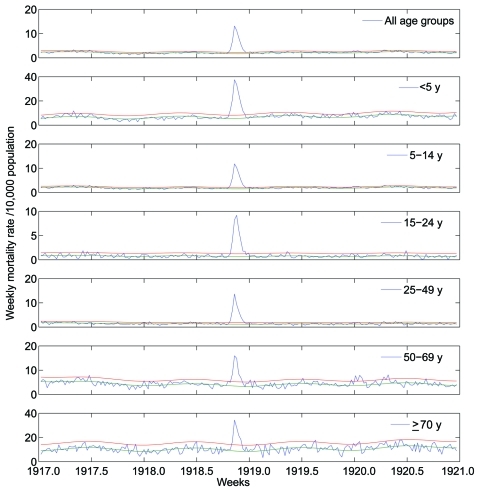
Age-stratified weekly all-cause mortality rates per 10,000 population in Boyacá, Colombia, 1917–1920. Background mortality rate derived from a seasonal regression model (blue); corresponding 95% CI curves are shown (red and green). Deaths in excess of the upper limit of the background mortality rate curve are deemed attributable to the 1918–19 influenza pandemic.

A total of 32,843 death records, written mostly by Catholic priests and corresponding to January 1917–December 1920, were manually retrieved from the parish archives of 78 municipalities in the department of Boyacá. From these archival records, we extracted age, cause, and exact date of death. To estimate mortality rates, we compiled weekly numbers of deaths from all causes and from respiratory illness, stratified into 5-year age groups (Figure 2, Figure 3). To obtain precise estimates of the transmission potential, we compiled daily death time series, combining all age groups.

#### Census Data

We obtained age-specific estimates of population size for the department of Boyacá from a 1918 census report (*15*). In 1918, ≈70% of Boyacá’s population was located in rural areas. During 1912–1918, the average annual population growth rate in Boyacá was 1.7%; during 1918–1920, it was 3.8%.

### Estimation of Excess Deaths

For characterization of mortality rates for the Boyacá pandemic, influenza-associated mortality rates must be separated from background mortality rates (deaths from respiratory illness other than influenza) and considered separately for each age group and cause of death (respiratory or all causes). To estimate pandemic mortality rate, we can define a discrete period of pandemic influenza activity and estimate the number of deaths in excess of background deaths that occurred during the pandemic period. Because mortality rates tend to vary seasonally throughout the year, our background estimate must also vary seasonally. To find the best estimate for baseline mortality rate in the absence of influenza activity, we applied regression methods, using harmonic terms and time trends, to mortality rate data (*6**,**16**,**17*) (Technical Appendix).

The regression model determines the extent to which observed weekly mortality rate fit the expectation of background mortality rate. Periods of poor fit indicate that observed mortality rate exceeds typical baseline levels, presumably because of increased influenza activity.

We defined pandemic periods as the weeks when deaths from respiratory illness exceeded the upper limit of the 95% CI of the background model. To estimate the mortality rate during the pandemic, for each age group we summed the weekly number of deaths from respiratory illness and from all causes that exceeded model baseline rates during each pandemic period during 1918–20.

To ensure that our estimates were not sensitive to modeling assumptions, we also estimated excess deaths by using an alternative approach to calculate background deaths. In this approach, background mortality rates for a given week are obtained by averaging mortality rates during the same week in previous years (Technical Appendix).

Finally, we estimated a relative measure of the effects of pandemic-associated deaths for each age group, which considers the typical mortality rate experienced by that age group. We calculated relative risk for pandemic-associated death, defined as the ratio of excess deaths during pandemic periods to expected baseline deaths. Relative risk has been shown to facilitate comparison between age groups or countries, which have different background risks for death (*17**,**18*).

### Comparing Patterns of Age-specific Deaths

We compared patterns of age-specific excess deaths from the 1918–19 Boyacá pandemic with those recently published for Mexico City (*3*). The estimates for Mexico City were based on excess-death rates obtained from monthly pneumonia and influenza records (1916–1920), stratified by 6 age groups (<5, 5–19, 20–29, 30–49, 50–69, and >70 years). Excess-death rates for Mexico City were calculated with a method similar to that used in this study.

We also reviewed key epidemiologic features of the pandemic in various locations as recently reported (*1**,**3**,**4**,**6**–**9**,**19**,**20*), focusing on comparisons of overall excess-death rates associated with the pandemic. We also reviewed epidemiologic evidence for early (herald) waves occurring before September 1918 and for death sparing among elderly persons. We limited the review to studies that provided monthly or weekly historical death data because such data enable identification of herald waves and precise estimation of excess-death rates.

### Estimation of Transmission Potential

Transmissibility of an infectious pathogen is measured by the basic reproduction number (R_0_), which is the average number of secondary infections generated by an infectious person in an entirely susceptible population (*21*). A related quantity is the reproduction number, R, which can be used for partially immune populations who have been vaccinated or previously exposed to similar pathogens (*21*).

We estimated R for the 1918 pandemic virus in Boyacá by using a simple method that relies on the epidemic growth rate, a measure of how fast the number of cases increases over time (Technical Appendix). Briefly, in the early ascending phase of an epidemic, the daily number of cases (or deaths) should follow an exponential function. By taking the log of daily deaths in the ascending phase, a straight line can be fit to the data. R can be derived from the growth rate estimate *r* by a simple equation involving the duration of the latency and infectious periods (*22*) (Technical Appendix).

Because of the uncertainty associated with duration of the latency and infectious periods for influenza, we considered periods of 1.5 and 2 days each (*23**,**24*). Latency and infectious periods can be summed into a single statistic called the generation interval, which measures the interval between disease onset in 2 successive cases. The generation intervals considered in this study were 3 and 4 days (*23**,**24*).

We defined the ascending phase as the period between the day of pandemic onset (defined as the first day of the period of steadily increasing deaths) and the day immediately before the epidemic peak. We tested the robustness of R estimates to the choice of death indicator (deaths from respiratory illness or from all causes). We also compared estimates derived from crude numbers of deaths and excess deaths from respiratory illness that were above background rates.

The same approach and assumptions have been used to quantify Rs associated with the 1918 pandemic in Copenhagen, Denmark, and Mexico City, and hence the Boyacá estimates are directly comparable to estimates from these studies (*3**,**6*). For comparison with Boyacá, we also reviewed the literature for published estimates of R associated with the 1918 pandemic in the Americas (*2**–**4*).

## Results

### Timing of Pandemic Waves and Age-specific Patterns of Death

**Figure 4:**
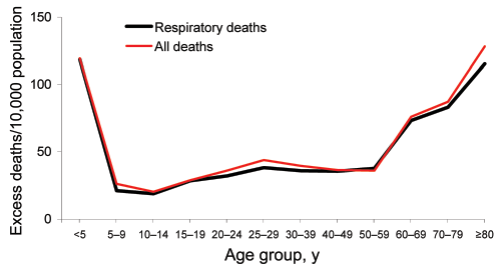
Age-specific excess-death rates per 10,000 population associated with the 1918–19 pandemic wave in Boyacá, Colombia, October 20, 1918, to January 26, 1919, based on deaths from respiratory illness and all causes.

The age-stratified time series of deaths from respiratory illness or all causes in Boyacá indicated that a severe pandemic wave occurred during a 15-week period, October 20, 1918–January 26, 1919 (Figure 2, Figure 3). The profile of age-specific excess deaths from respiratory illness associated with the pandemic period formed a W-shaped pattern; peak mortality rates among infants (<5 years of age) were followed by peak rates among elderly persons (>60 years) and young adults (25–29 years) (Table 1). Excess deaths were lowest among children 5–14 years of age and adults 50–59 years of age. Similar age patterns were found for all-cause deaths (Figure 4); the correlation coefficient between respiratory and all-cause excess-death rates was >0.99 (p<0.01). Excess deaths from respiratory illness captured most influenza-related all-cause excess deaths across all age groups (95% on average, range 81%–100%). Confidence intervals were larger for the most extreme age groups.

**Table 1:** Age-specific excess-death rates associated with the October 1918 –January 1919 influenza pandemic wave in Boyacá, Colombia*

Age group, y	Deaths from respiratory illness			Deaths from all causes	
	Excess mortality rate/10,000 population (95% CI)	Relative risk/ background mortality rate†		Excess mortality rate/10,000 population (95% CI)	Relative risk/ background mortality rate†
All ages	40.1 (39.1–41.1)	5.2		42.1 (39.1–44.1)	1.7
0–4	118.1 (111.1–125.1)	3.0		118.1 (109.1–127.1)	1.3
5–9	21.1 (20.1–23.1)	13.5		26.1 (23.1–29.1)	3.6
10–14	19.1 (18.1–20.1)	11.4		18.1 (16.1–20.1)	3.2
15–19	28.1 (27.1–30.1)	13.4		27.1 (24.1–30.1)	3.3
20–24	32.1 (30.1–33.1)	12.6		35.1 (31.1–38.1)	3.5
25–29	36.1 (34.1–37.1)	51.3		42.1 (39.1–45.1)	5.7
30–39	37.1 (35.1–38.1)	6.9		39.1 (36.1–42.1)	2.2
40–49	36.1 (33.1–39.1)	11.8		36.1 (31.1–41.1)	1.6
50–59	35.1 (31.1–40.1)	11.5		27.1 (19.1–35.1)	1.3
60–69	73.1 (67.1–80.1)	4.1		69.1 (55.1–82.1)	1.1
70–79	83.1 (69.1–98.1)	3.4		82.1 (59.1–106.1)	0.9
>80	100.1 (81.1–120.1)	3.5		124.0 (87.2–160.8)	0.9

*Excess death estimates are based on observed mortality rates during pandemic weeks occurring in excess of background mortality rates derived from a seasonal regression model. †Ratio of excess deaths divided by background deaths during influenza pandemic weeks, facilitating comparisons across age groups with different background risks for death.

To facilitate the comparison between population age groups with different background risks for death, we calculated the risk for excess-death rates relative to baseline rates (Table 1). Although absolute excess-death rates were highest for young children (0–4 years of age) and elderly persons (>60 years), during the pandemic the relative risks were lowest for these age groups. Relative risk was highest for young adults 25–29 years of age; excess-death rates increased 51-fold above background death rates for respiratory causes and 6-fold for all causes.

**Figure 5:**
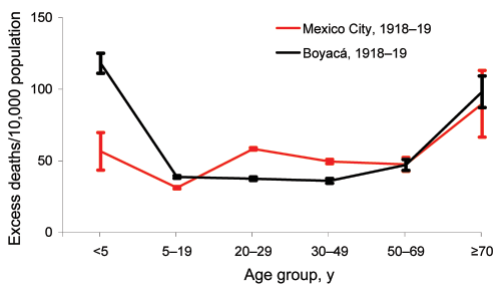
Comparison of age-specific excess-death rates for respiratory diseases during the main wave of the 1918–19 influenza pandemic in Mexico City, Mexico, and Boyacá, Colombia. Error bars represent 95% CIs.

Comparison of Boyacá and Mexico City shows that age-specific excess-death rates produced a W-shaped pattern for both locations (Figure 5). However, excess-death rates among young adults (20–29 years) were substantially higher for Mexico City than for Boyacá. By contrast, excess-death rates among infants were 2-fold lower for Mexico City than for Boyacá. Excess-death rates for elderly persons were similar for both cities. Overall, we estimate that the October 1918–January 1919 pandemic period was associated with 47 and 40 excess respiratory deaths per 10,000 population in Mexico City and Boyacá, respectively.

A broader comparison of epidemiologic patterns associated with the pandemic at 12 locations on different continents highlights substantial variations in the timing, number of pandemic waves, and age-specific death rates (Table 2). Europe and the United States generally experienced herald waves in during March–August 1918 (except for Paris) and low excess-death rates among elderly populations. In contrast, there was no evidence of death sparing among elderly populations in Latin America or Asia, and herald waves occurred at 4 of the 7 locations studied in these regions. Excess-death rates from respiratory illness were high for Iquitos, Peru; Toluca, Mexico; and Basque Provinces, Spain (121–288 deaths/10,000 population); intermediate in Taiwan (78–180 deaths/10,000 population); and lower elsewhere, including in Boyacá (29–67 deaths/10,000 population).

**Table 2:** Main epidemiologic features of the 1918–1920 influenza pandemic*

Location	Herald wave in 1918	Excess mortality rate from respiratory illness/10,000 population, main 1918–19 wave (mo of peak pandemic deaths, 1918)	Death-sparing effect among elderly persons	Reference
Americas				
New York, USA	Yes (Mar–Apr)	52 (Oct–Nov)	Yes	(*1*)
Mexico City, Mexico	Yes (May)	47 (Nov)	No	(*3*)
Toluca, Mexico	Yes (May)	162 (Nov)	No	(*3*)
Boyacá, Colombia	No	40 (Nov)	No	This study
Lima, Peru	Yes (Sep–Oct)†	29 (Nov)	No†	(*4*)
Iquitos, Peru	No	288 (Nov)	ND	(*4*)
Europe				
Copenhagen, Denmark	Yes (Jul–Aug)	39 (Nov)	Yes	(*6*)
Paris, France	No	61 (Oct)	ND	(*7*)
Basque Provinces, Spain	Yes (Jun)	121 (Oct)	ND	(*19*)
Madrid, Spain	Yes (Jun)	53 (Oct)	Yes	(*7*)
Asia				
Taiwan	No	67 (Nov)	No	(*8*,*20*)
Singapore	Yes (Jul)	78–180 (Oct)	ND	(*9*,*20*)

*Data from quantitative studies across different locations around the world. Locations are organized by continent (America, Europe, Asia) and latitude. ND, not determined. †Cannot conclude because of lack of age-specific population data.

### Reproduction Number Estimates

Table 3 provides summary estimates for the R for the 1918 influenza pandemic in Boyacá, based on growth in daily rates for death from respiratory illness. R was estimated to be 1.4, assuming a short generation interval of 3 days, and 1.5–1.6, assuming a longer interval of 4 days. A sensitivity analysis, based on excess deaths from respiratory illness occurring above a background of expected deaths, generated slightly higher R estimates (1.4–1.5 for a generation interval of 3 days and 1.6–1.7 for a generation interval of 4 days). Different approaches for estimating background deaths resulted in R estimate differences of <0.06 (4%).

**Table 3:** Estimates of the growth rate and reproduction number associated with the 1918–19 influenza pandemic in Boyacá, Colombia*

Mortality outcome	Early growth phase period, 1918	Daily growth rate, mean (95% CI)	R estimate, mean (95% CI)				
			3-d generation interval			4-d generation interval	
			Exp dist.	Delta dist.		Exp dist.	Delta dist.
Deaths from respiratory illness	Oct 13–Nov 15	0.121 (0.120–0.122)	1.40 (1.39–1.40)	1.44 (1.43–1.44)		1.54 (1.54–1.54)	1.62 (1.62–1.63)
Excess deaths from respiratory illness	Oct 27–Nov 15	0.137 (0.136–0.139)	1.45 (1.45–1.46)	1.51 (1.51–1.52)		1.62 (1.62–1.63)	1.73 (1.72–1.74)

*Estimates are based on daily data. A generation interval of 3 or 4 d is assumed, with an exponential (exp) or a fixed (delta) distribution (dist.). R, reproduction number.

Comparison of estimates derived from different locations in the Americas revealed some geographic variations in the transmission potential of the 1918–19 pandemic wave (Table 4). Although R estimates were 1.3–1.8 in most locations in the Americas, assuming a 3-day generation interval, the transmissibility of influenza during the autumn wave might have been particularly high in Toluca, Mexico (estimated R = 2.0–2.5).

**Table 4:** Estimates of the reproduction number across influenza pandemic locations in the Americas , 1918–19*

Location, north to south	Time of pandemic wave	R estimate		Source
		3-d serial interval	6-d serial interval	
45 US cities†	1918 autumn†	1.7–1.8	2.5–3.3	(*2*,*22*)
Toluca	1918 spring	1.6–1.8	2.4–3.1	(*3*)
Toluca	1918 autumn	2.0–2.5	3.2–6.1	(*3*)
Mexico City	1918 spring	1.3–1.3	1.7–1.8	(*3*)
Mexico City	1918 autumn	1.3–1.3	1.6–1.7	(*3*)
Boyacá, Colombia	1918 Oct–Nov	1.4–1.5	1.8–2.3	This study
Lima, Peru	1918 Nov–1919 Feb	1.3–1.4	1.6–2.0	(*4*)

*Values are based on a range of estimates provided by considering different distributions of the generation interval (exponentially distributed latent and infectious periods or fixed generation interval). R, reproduction number. †R estimates are based on the mean of the initial growth rates across 45 US cities.

## Discussion

Our study makes use of extensive archival death records covering before and during the 1918–19 influenza pandemic in Boyacá, Colombia, and confirms the substantial number of deaths caused by the pandemic in this region. The main epidemiologic features of the pandemic in Boyacá include a single wave of excess deaths during October 1918–January 1919; high excess-death rates among infants and elderly persons; and a moderate R (estimated at 1.4–1.5, assuming a 3-day generation interval).

We did not identify a herald wave of deaths from pandemic influenza in the early part of 1918 in Boyacá. According to epidemiologic data, herald waves of mild pandemic activity have been reported for the spring and summer of 1918 in other regions of the world, including New York City (*1*), Mexico (*3*), Lima (*4*) Geneva (*25**,**26*), Copenhagen (*6*), military camps in the United States (*6*), the United Kingdom (*27*), and Singapore (*9*). The absence of a herald wave in Boyacá could be explained by late introduction of the pandemic influenza virus; alternatively, a mild first wave may have occurred without causing many deaths. Thus, we cannot rule out early pandemic activity, which might have been associated with mild illnesses, before October 1918 in Boyacá. For instance, the summer pandemic wave of 1918 in Denmark was clearly evident only from time-series case data (*6*). These epidemiologic findings suggesting early pandemic virus activity have recently been confirmed by sequencing of pandemic influenza virus specimens isolated from Army camp populations in the United States as early as May 1918 (*28*).

Although substantial postpandemic waves have been reported for 1919–20 in New York City (*1*), Mexico City (*3*), Lima (*4*), Japan (*5*), and Taiwan (*8*), we could not identify a clear recrudescent pandemic wave in 1920 in Boyacá. A 3-week period in January 1920 and a 4-week period in April–May 1920 were associated with a small increase in deaths from respiratory illness, mostly affecting elderly persons, but we cannot with certainty attribute these deaths to pandemic influenza. Early public health warnings and effective implementation of control interventions in large cities such as New York City, Mexico City, Lima, and Taiwan, could have contributed to maintaining a large pool of susceptible persons, which could fuel subsequent pandemic waves (*29*). In Japan, postpandemic waves were somewhat limited to regions that escaped earlier waves (*5*). Given that Boyacá was a relatively small rural area, pandemic activity in 1918 might have proceeded unabated, with no particular interventions, medical or nonmedical. Alternatively, Boyacá could have escaped the recrudescent pandemic wave in 1920 because of its remote location. Overall, the main wave of deaths from pandemic influenza that occurred during October 1918–January 1919 in Boyacá is reminiscent of the single wave of pandemic influenza A (H1N1) 2009 wave that occurred in the Southern Hemisphere during the winter of 2009 (e.g., Chile [*30*], Australia [*31*], and New Zealand [*31*]). Additional data from the 1918 pandemic in other Southern Hemisphere locations are warranted before these findings can be generalized.

The W-shaped age-specific pattern of deaths during the 1918–19 pandemic wave in Boyacá is in agreement with recent reports from the Mexico City area (*3*) and Peru (*4*). These reports suggest a lack of death sparing among elderly populations of urban and rural areas of Latin America, although data from additional locations would be useful for generalizing these conclusions. This pattern is also in agreement with anecdotal evidence from aboriginal populations in Alaska in 1918 (*32*). In contrast to reports for Latin America and Alaska, reports for the United States and Europe suggest that elderly populations were substantially protected from influenza-associated death in 1918 (*1**,**5**,**6*). Previous studies have hypothesized that childhood exposure to influenza A (H1N1) viruses before 1870 might account for prior immunity among elderly persons during the 1918 pandemic. A similar phenomenon has been noted for pandemic (H1N1) 2009, during which risk for clinical infection and death was lower during the pandemic than during seasonal epidemics for persons >60 years of age (*13**,**33*).

Regional differences in prior immunity to influenza might result from heterogeneous circulation of influenza viruses during the 19th century, when long-distance travel was much less common than it is today (*3*). In 1918, Colombia’s population of 5.8 million was heterogeneously distributed and relatively isolated from the rest of the world (*34*); this isolation could explain the lack of exposure to influenza viruses during the middle of the 19th century. Also in 1918, transportation was underdeveloped in Colombia, consisting mostly of horse- or mule-drawn street cars, waterways, and sparse railroads that did not connect with Boyacá (*34*). Remoteness could have affected the probability of introduction and of local dissemination of influenza viruses in the Boyacá region. A similar phenomenon could also explain the apparent lack of a herald pandemic wave in the spring of 1918, when pandemic virus activity was not yet globally widespread. Of note, the capital city, Bogota, was the first area in Colombia to report increased influenza activity in October 1918; the virus quickly spread to other Colombia locations (*34*).

Excess-death rates among young adults were lower in Boyacá than in Mexico City (*3*). The reasons for this difference are unclear but could be associated with a more sporadic distribution of the population in Boyacá, resulting in lower overall influenza attack rates; however, we do not have epidemiologic evidence to support this assumption. Alternatively, the unidentified factors that made young adults particularly susceptible to influenza-related death in Europe, the United States, and Mexico in 1918 (*1**,**5**,**6*) might have been less common among young adults in Colombia. Despite these geographic differences in absolute risk for death from pandemic influenza, in all locations with sufficient data the relative risk for death consistently peaked among adults 20–29 years of age when compared with baseline death rates during nonpandemic years. Hence, our study confirms the universal atypical severity of this virus in young adults, as previously reported for the United States (*1*), Mexico (*3*), Europe (*6**,**7*), and Taiwan (*8*). We also note that data from Boyacá and Mexico City do not support the pessimistic hypothesis that populations lacking prior immunity to the 1918 virus would experience a V-shaped age-associated risk for death, in which risk would rapidly and continuously rise past teenage years (*35*).

Our excess-deaths approach warrants some caveats. The regression model used to estimate background deaths poorly fit the Boyacá data during the nonpandemic period, probably because of weak seasonality. However, our estimates of excess deaths from pandemic influenza based on deaths from respiratory illness and all causes were highly correlated, similar to those from other temperate countries, where baseline death rates are more seasonal (*1**,**3**,**6*). The sensitivity analysis that we conducted by using an alternative approach to estimate background deaths did not make assumptions about seasonality (*20*). This analysis produced excess-death estimates highly correlated with those derived from the regression approach (correlation = 0.97; p<0.01; mean difference 4%–7%).

Transmissibility estimates derived from 1918–20 pandemic illness and death data are 1.5–5.4 for community-based settings in several regions of the world (*2**,**6**,**36**,**37*) (Table 4). Our transmissibility estimates for Boyacá, Colombia, assuming a generation interval of 3 days, are in close agreement with those reported for the wave in autumn in Mexico City (*3*), Lima (*4*), England and Wales (*27*), and Copenhagen (*6*) and slightly lower than estimates reported for the city of Toluca, Mexico (*3*), and US cities (*2**,**38*). Boyacá’s sparsely distributed population could explain why the estimated disease transmissibility is relatively low. It remains unclear whether differences in reproduction number estimates across locations and pandemic waves reflect true differences attributable to variation in attack rates or local factors affecting transmission or merely illustrate difficulties in measuring this parameter with precision (*38*). In previous studies focused on reproduction number estimates in which we used similar data and approaches, we have shown that inclusion of a delay between disease onset and death has little effect on the estimates (*39*).

In conclusion, historical studies from understudied areas are especially helpful for documenting the global death rates and transmission patterns of the 1918 pandemic and for revealing substantial variations among locations. In particular, the lack of death sparing for elderly persons in Colombia and Mexico differs markedly from contemporaneous observations in the United States and Europe. During the 19th century, the Latin American region was relatively isolated (and still is today) (*40*), which would affect the circulation of historical influenza viruses and baseline population immunity to influenza. We believe that this finding suggests recycling of influenza viruses as the best explanation for death sparing among elderly persons in the United States and Europe in 1918. Preservation and interpretation of archival epidemiologic data are crucial for a better understanding of past pandemics and for better preparedness against future pandemics.

## Supplementary Material

Technical Appendix To estimate the mortality attributable to the influenza pandemic, we calculated mortality rate in excess of a seasonal model baseline and occurring during pandemic activity periods...
