# Supplementary material for: The 1918–19 Influenza Pandemic in Boyacá, Colombia
Source: Emerg Infect Dis. 2012 Jan;18(1):48–56. doi: 10.3201/eid1801.101969 (PMC3310082; doi:10.3201/eid1801.101969)
Supplement: Technical Appendix — To estimate the mortality attributable to the influenza pandemic, we calculated mortality rate in excess of a seasonal model baseline and occurring during pandemic activity periods... [file 10-1969-Techapp_4p.pdf]

Article DOI: http://dx.doi.org/10.3201/eid1801.101969

The 1918–19 Influenza Pandemic in Boyacá, Colombia

Technical Appendix

Excess Mortality Models

To estimate the mortality attributable to the influenza pandemic, we calculated mortality rate in excess of a seasonal model baseline and occurring during pandemic activity periods, using weekly time series of respiratory and all-cause mortality rates (*1–3*). We established the seasonal baseline by applying a cyclical Serfling linear regression model to weekly death rates, after excluding data from October 1918–January 1919. Specifically, the model can be written as:

$$\text{weekly death rates}(t) = \text{intercept} + \alpha \times t + \beta * \sin(2 \times \pi/52.17 \times t) + \gamma * \cos(2 \times \pi/52.17 \times t)$$

where t is the week number and α, β, and γ are coefficients to be estimated from the data, representing the time trend and seasonal oscillations, respectively. Influenza epidemic periods were defined as the weeks when mortality exceeded the upper limit of the 95% CI on this baseline (Technical Appendix Figure). We summed the excess deaths above the model baseline during each epidemic period identified during 1918–1920 to estimate the mortality rate of the pandemic. Given that there is little apparent seasonality in Colombian respiratory and all-cause mortality data (Figures 2, 3 in main text), we fitted stepwise regression models separately for each age group and outcomes to select covariates for time trends and seasonality. Given the lack of seasonality, the model explained a low fraction of the variance in data ($R^2<0.20$), except for extreme age groups ($R^2 \approx 0.5$).

As a sensitivity analysis, we also estimated excess mortality using a model-free approach, in which reference months in prepandemic years are used to estimate baseline mortality (adapted from [*4*]).

Estimation of transmission characteristics (reproduction number)

The basic reproduction number (R_0) is defined as the average number of secondary cases generated by a primary case at the onset of an epidemic in an entirely susceptible population (*5,6*). A related quantity is the reproduction number, R, which captures partial immunity in the population due to previous exposure of the population to related influenza viruses or vaccination campaigns (*7*). We estimated the reproduction number, R, using the intrinsic growth rate method, as in (*7,8*). We estimated the growth rate r by fitting an exponential function to the initial increase in the daily number of excess respiratory deaths (*9*). By taking the log of daily deaths in the ascending phase, a straight line can be fit to the data. Specifically, the regression can be written as log(daily cases(t)) = intercept + $r \times t$, where t is a daily index, and r is a regression coefficient to be estimated, representing the exponential growth rate. The early ascending phase was determined as the period between the day of pandemic onset, defined as the first day of the period of monotonously increasing deaths, and the day immediately preceding the epidemic peak. The reproduction number was calculated by substituting the estimate for r into an expression derived from the linearization of the classical Susceptible-Exposed-Infectious-Recovered transmission model (*8,10*):

$$R=(1+\frac{r}{b_1})(1+\frac{r}{b_2}) \qquad (1)$$

where $1/b_1$ and $1/b_2$ are respectively the mean latent and infectious periods. This expression for R assumes exponentially distributed latent and infectious periods where the mean generation interval between two successive cases is given by $T_c = 1/b_1 + 1/b_2$. To assess the impact of the distribution of the generation interval on the reproduction number, we also obtained an upper bound estimate for the extreme case of a fixed generation interval (delta distribution), using the following expression (*8*):

$$R = e^{rT_c} \qquad (2)$$

We also tested the robustness of R estimates to the choice of mortality indicator and compared estimates derived from crude respiratory deaths and respiratory deaths in excess of the model baseline.

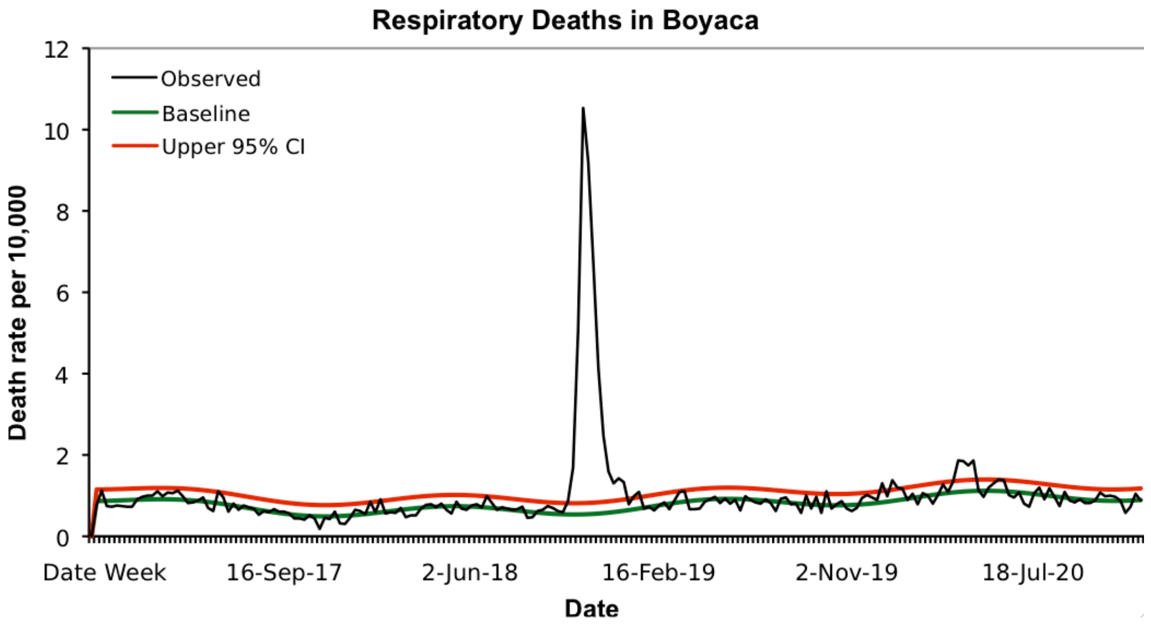

Technical Appendix Figure. Daily time series of respiratory deaths in Boyaca, Colombia from 1917 to 1920. Excess deaths are above the upper limit of the baseline mortality curve calibrated using mortality levels prior to the 1918 influenza pandemic.

References

1. Serfling RE. Methods for current statistical analysis of excess pneumonia-influenza deaths. Public Health Rep. 1963;78:494–506. PubMed doi:10.2307/4591848

2. Viboud C, Grais RF, Lafont BA, Miller MA, Simonsen L. Multinational impact of the 1968 Hong Kong influenza pandemic: evidence for a smoldering pandemic. J Infect Dis. 2005;192:233–48. PubMed doi:10.1086/431150

3. Andreasen V, Viboud C, Simonsen L. Epidemiologic characterization of the 1918 influenza pandemic summer wave in Copenhagen: implications for pandemic control strategies. J Infect Dis. 2008;197:270–8. PubMed doi:10.1086/524065

4. Murray CJ, Lopez AD, Chin B, Feehan D, Hill KH. Estimation of potential global pandemic influenza mortality on the basis of vital registry data from the 1918-20 pandemic: a quantitative analysis. Lancet. 2006;368:2211–8. PubMed doi:10.1016/S0140-6736(06)69895-4

5. Anderson RM, May RM. Infectious diseases of humans. Oxford: Oxford University Press; 1991.

6. Diekmann O, Heesterbeek J. Mathematical epidemiology of infectious diseases: model building, analysis and interpretation: Wiley; 2000.

7. Chowell G, Bettencourt LM, Johnson N, Alonso WJ, Viboud C. The 1918-1919 influenza pandemic in England and Wales: spatial patterns in transmissibility and mortality impact. Proc Biol Sci. 2008;275:501–9. PubMed doi:10.1098/rspb.2007.1477

8. Wallinga J, Lipsitch M. How generation intervals shape the relationship between growth rates and reproductive numbers. Proc Biol Sci. 2007;274:599–604. PubMed doi:10.1098/rspb.2006.3754

9. Chowell G, Nishiura H, Bettencourt LM. Comparative estimation of the reproduction number for pandemic influenza from daily case notification data. J R Soc Interface. 2007;4:155–66. PubMed doi:10.1098/rsif.2006.0161

10. Lipsitch M, Cohen T, Cooper B, Robins JM, Ma S, James L, et al. Transmission dynamics and control of severe acute respiratory syndrome. Science. 2003;300:1966–70. PubMed doi:10.1126/science.1086616
